# Efficient and Reabsorption‐Free Radioluminescence in Cs_3_Cu_2_I_5_ Nanocrystals with Self‐Trapped Excitons

**DOI:** 10.1002/advs.202000195

**Published:** 2020-04-16

**Authors:** Linyuan Lian, Moyan Zheng, Weizhuo Zhang, Lixiao Yin, Xinyuan Du, Peng Zhang, Xiuwen Zhang, Jianbo Gao, Daoli Zhang, Liang Gao, Guangda Niu, Haisheng Song, Rong Chen, Xinzheng Lan, Jiang Tang, Jianbing Zhang

**Affiliations:** ^1^ School of Optical and Electronic Information Huazhong University of Science and Technology 1037 Luoyu Road Wuhan Hubei 430074 China; ^2^ Wuhan National Laboratory for Optoelectronics Huazhong University of Science and Technology Wuhan Hubei 430074 China; ^3^ College of Physics and Optoelectronic Engineering Shenzhen University Shenzhen 518060 China; ^4^ Ultrafast Photophysics of Quantum Devices Department of Physics and Astronomy Clemson University Clemson SC 29634 USA; ^5^ State Key Laboratory of Digital Manufacturing Equipment and Technology School of Mechanical Science and Engineering Huazhong University of Science and Technology Wuhan Hubei 430074 China; ^6^ Shenzhen Huazhong University of Science and Technology Research Institute Shenzhen Guangdong 518057 China

**Keywords:** cesium copper halide, nanocrystals, radioluminescence, scintillators

## Abstract

Radioluminescent materials (scintillators) are widely applied in medical imaging, nondestructive testing, security inspection, nuclear and radiation industries, and scientific research. Recently, all‐inorganic lead halide perovskite nanocrystal (NC) scintillators have attracted great attention due to their facile solution processability and ultrasensitive X‐ray detection, which allows for large area and flexible X‐ray imaging. However, the light yield of these perovskite NCs is relatively low because of the strong self‐absorption that reduces the light out‐coupling efficiency. Here, NCs with self‐trapped excitons emission are demonstrated to be sensitive, reabsorption‐free scintillators. Highly luminescent and stable Cs_3_Cu_2_I_5_ NCs with a photoluminescence quantum yields of 73.7%, which is a new record for blue emission lead‐free perovskite or perovskite‐like NCs, is produced with the assistance of InI_3_. The PL peak of the Cs_3_Cu_2_I_5_ NCs locates at 445 nm that matches with the response peak of a silicon photomultiplier. Thus, Cs_3_Cu_2_I_5_ NCs are demonstrated as efficient scintillators with zero self‐absorption and extremely high light yield (≈79 279 photons per MeV). Both Cs_3_Cu_2_I_5_ NC colloidal solution and film exhibit strong radioluminescence under X‐ray irradiation. The potential application of Cs_3_Cu_2_I_5_ NCs as reabsorption‐free, low cost, large area, and flexible scintillators is demonstrated by a prototype X‐ray imaging with a high spatial resolution.

## Graphical abstract

Nanocrystals (NCs) with self‐trapped excitons emission are demonstrated to be sensitive, reabsorption‐free scintillators. Highly blue‐emissive and stable Cs_3_Cu_2_I_5_ NCs with a photoluminescence quantum yield of 73.7% are produced. These Cs_3_Cu_2_I_5_ NCs show a strong and reabsorption‐free radioluminescence under X‐ray irradiation with an extremely high light yield, which makes them promising scintillators for low cost, large area, and flexible X‐ray imaging.

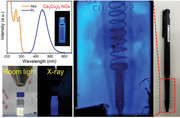

## 1 Introduction

Scintillators have actively been used for radiation detection in the fields of medical imaging, nondestructive testing, security inspection, and nuclear power stations, owing to their intrinsic ability to absorb high energy X‐ray photons and convert into visible light.^[^ ^1^, ^2^, ^3^, ^4^, ^5^ ^]^ X‐ray detectors based on scintillators are widely adopted in the commercial market, because they are much cheaper and more stable than direct‐type detectors that directly convert X‐ray photons to electrical signals by applied bias voltage in photoconductors.^[^ ^6^, ^7^, ^8^, ^9^, ^10^, ^11^, ^12^ ^]^ Currently, conventional scintillators, such as thallium‐doped cesium iodide (CsI:Tl) and cerium‐doped lutetium yttrium orthosilicate (LYSO:Ce), are typically fabricated by crystallization under high temperature and vacuum conditions, which is complicated and expensive.^[^ ^13^ ^]^ The resulting bulk crystals are inherently brittle and fragile, which severely restrict their application in flexible detection like dental and oral radiography.^[^ ^6^, ^14^ ^]^ Additionally, the application of crystalline scintillators is limited for large area X‐ray imaging due to the difficulty in producing large area crystalline materials. Therefore, development of cost‐effective, low temperature, and solution‐processable scintillators is highly desirable.

Recently, all‐inorganic lead halide perovskite nanocrystals (NCs) have emerged as a new generation of scintillating materials and exhibit a lot of exciting radiation detection properties, such as large stopping power, strong and tunable radioluminescence (RL), fast scintillation response, ultrasensitive X‐ray sensing, and high‐resolution imaging.^[^ ^6^, ^14^, ^15^, ^16^, ^17^ ^]^ Although these NCs exhibit very high photoluminescence quantum yield (PLQY) of up to 90%, the RL light yield of CsPbBr_3_ NCs is only 21 000 photons per MeV.^[^ ^16^ ^]^ Such value is inferior to that of conventional scintillators like CsI:Tl (54 000 photons per MeV) and LYSO:Ce (33 200 photons per MeV).^[^ ^18^, ^19^ ^]^ The relatively low light yield of lead halide perovskite NCs is attributed to the intrinsic small Stokes shift and strong self‐absorption effect, which could substantially reduce the light out‐coupling efficiency.^[^ ^19^, ^20^ ^]^ Therefore, a large Stokes shift, small self‐absorption and a high PLQY are essential for a high RL light yield.^[^ ^19^ ^]^ In addition, the high toxicity and bioaccumulation of lead component in lead halide perovskite NCs is a potential issue that limits their wide spread applications. Thus, developing high PLQY, large Stokes shift, and lead‐free or eco‐friendly scintillating NCs are of great significance.

Here, we demonstrate that colloidal NCs with self‐trapped excitons (STEs) emission can be efficient scintillators due to a high PLQY and large Stokes shift. Recently, Cs_3_Cu_2_ *X*_5_ (*X* = Cl, Br, I) have been shown to be highly luminescent perovskite‐like materials with high PLQYs and large Stokes shifts.^[^ ^21^, ^22^, ^23^, ^24^, ^25^ ^]^ Moreover, colloidal NCs of these materials were also developed. For example, Han et al. produced Cs_3_Cu_2_I_5_ NCs with a PLQY of 67%, however the stability of their PLQY was not reported.^[^ ^26^ ^]^ Quan et al. synthesized stable Cs_3_Cu_2_I_5_ NCs with the PLQY maintained for 45 days in air, however, the PLQY is relatively low (29.2%).^[^ ^27^ ^]^ In the present work, we synthesized highly luminescent and stable Cs_3_Cu_2_I_5_ NCs using a modified hot injection method with the assistance of indium iodide (InI_3_). The addition of InI_3_ is critical for our synthesis, because it allows the synthesis to be performed at a relatively high temperature which renders the Cs_3_Cu_2_I_5_ NCs a high PL QY and high stability in air. The Cs_3_Cu_2_I_5_ NCs exhibit a reabsorption‐free, highly efficient, and stable blue emission peaked at 445 nm with a PLQY of up to 73.7%. Such high PLQY is a new record for blue emission lead‐free perovskite or perovskite‐like NCs at present. Additionally, no noticeable decrease of the PLQY was observed in 30 days of air‐storage. The appealing advantages of Cs_3_Cu_2_I_5_ NCs include highly emissive STEs due to 0D electronic structure and a large Stokes shift that is beneficial for efficient light out‐coupling. The high PLQY, zero self‐absorption, large Stokes shift, and high stopping power combined with emission spectrum matching quite well with response peak of a silicon photomultiplier (SiPM) make Cs_3_Cu_2_I_5_ NCs ideal scintillators with an extremely high light yield of 79 279 photons per MeV. Additionally, we showed the promising application of X‐ray imaging of Cs_3_Cu_2_I_5_ NCs by our prototype imaging with a high resolution of 0.32 mm. We believe that their unique optical properties render great potential for Cs_3_Cu_2_I_5_ NCs to be outstanding candidates as novel and high‐performance scintillators.

## 2 Results and Discussion

### 2.1 Synthesis of Cs_3_Cu_2_I_5_ NCs

Colloidal Cs_3_Cu_2_I_5_ NCs were synthesized using a modified hot‐injection method,^[^ ^26^, ^27^ ^]^ in which metal halides CuI and InI_3_ are first dissolved in organic solvent octadecene together with oleic acid (OA) and oleylamine (OLA) ligands, then the solution was heated to 180 °C and the nucleation and growth of Cs_3_Cu_2_I_5_ NCs is then triggered by the swift injection of Cs‐oleate. After 30 s, the reaction mixture was cooled to room temperature using an ice‐water bath, and the Cs_3_Cu_2_I_5_ NCs were separated by centrifugation (see the experimental section for the details). It is worth mentioning that the addition of InI_3_ is essential for high PLQYs, which is the main difference compared to recently reported synthesis of Cs_3_Cu_2_I_5_ NCs.^[^ ^26^ ^]^ We found that high injection and growth temperatures are favorable for high PLQYs either with or without the presence of InI_3_. This is in agreement with the well‐known rule that a high temperature is beneficial for crystallization and generally produces less defects. However, we found, without the addition of InI_3_, the Cs_3_Cu_2_I_5_ NCs grew too large and lost colloidal stability in solvents (Figure S1, Supporting Information). The InI_3_ is capable of inhibiting the growth of Cs_3_Cu_2_I_5_ NCs, thus it is possible to produce Cs_3_Cu_2_I_5_ NCs with colloidal stability and high PLQYs at a high temperature, such as 180 °C. Additionally, we found the Cs_3_Cu_2_I_5_ NC size decreases with increasing amount of InI_3_, as shown in Figures S2 and S3, Supporting Information. However, the In^3+^ is not incorporated into the lattice of Cs_3_Cu_2_I_5_ based on the following powder X‐ray diffraction (XRD) and elemental analysis. The role of InI_3_ is similar with that of zinc halide salt which was introduced to precisely control the size of CsPbBr_3_ NCs.^[^ ^28^, ^29^ ^]^

**Figure 1:**
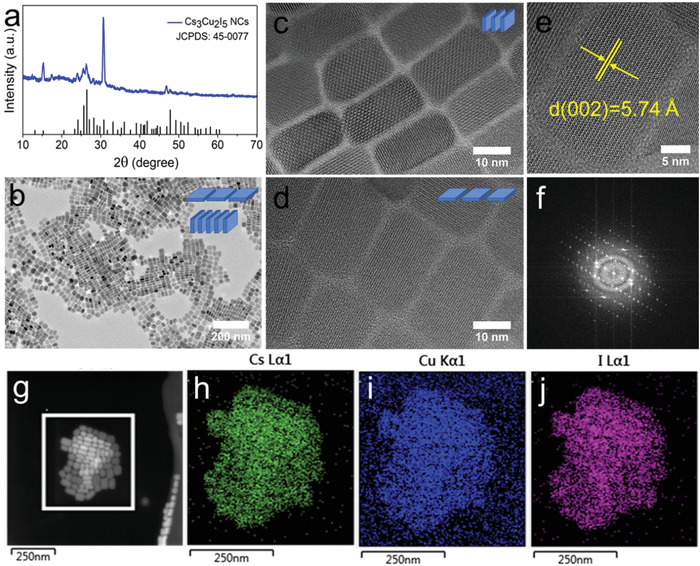
a) XRD pattern and b) TEM image of Cs_3_Cu_2_I_5_ NCs. HRTEM images with the Cs_3_Cu_2_I_5_ NCs c) in face‐to‐face stacking and d) lying flat. e) A typical HRTEM image of a single Cs_3_Cu_2_I_5_ NC with enlarged view, and f) its corresponding FFT pattern. g) HAADF‐STEM measurement of Cs_3_Cu_2_I_5_ NCs, and the corresponding EDS mapping of elemental h) Cs, i) Cu, and j) I distributions.

The crystal phase of Cs_3_Cu_2_I_5_ NCs was confirmed by the powder XRD. XRD pattern of Cs_3_Cu_2_I_5_ NCs is well coincident with the bulk counterpart as shown in **Figure** **1**a, without secondary crystalline phases. The Cs_3_Cu_2_I_5_ NCs adopt an orthorhombic space group Pnma with lattice parameters of *a* = 10.1743 Å, *b* = 11.6532 Å, and *c* = 14.3621 Å.^[^ ^21^ ^]^ In these materials, isolated [Cu_2_I_5_]^3−^ clusters are surrounded by the Cs^+^ cations to form a unique 0D structure at the molecular level. Such unique 0D structure will exhibit remarkable and useful properties.^[^ ^28^ ^]^ The morphology and size distribution of Cs_3_Cu_2_I_5_ NCs were further characterized by transmission electron microscopy (TEM). As shown in Figure 1b, the TEM image of Cs_3_Cu_2_I_5_ NCs shows a brick shape with lying flat and standing vertical to the substrate. Unlike spherical NCs, the size and size distribution of the Cs_3_Cu_2_I_5_ NCs were determined by three characteristic parameters: *l*, side length; *w*, side width; *h*, height for the nanobricks. The size and size distribution are shown in Figure S4, Supporting Information, demonstrating uniformity of the Cs_3_Cu_2_I_5_ NCs. As shown in Figure 1c, self‐assembled nanobricks stacked in a face‐to‐face manner, which allowed us to measure the thickness precisely. The thickness of the nanobricks is around 10 nm. Figure 1d shows nanobricks lying flat on the TEM grid, the length and width of the nanobricks are around 30 and 20 nm, respectively. High‐resolution TEM (HRTEM) (Figure 1e) and its fast Fourier transform (FFT) (Figure 1f) of a single Cs_3_Cu_2_I_5_ nanobrick confirm the high crystallinity without obvious crystal defects. The clear interplanar distance of 5.74 Å corresponds to the (002) crystal face. Moreover, their high‐angle annular dark‐field scanning TEM (HAADF‐STEM) image (Figure 1g) and corresponding elemental mapping (Figure 1h–j) demonstrate the uniform distributions of cesium (Cs), copper (Cu), and iodine (I) elements in the Cs_3_Cu_2_I_5_ NCs. In order to confirm that the In^3+^ ions are not incorporated into the lattice of the as‐prepared Cs_3_Cu_2_I_5_ NCs, quantitative energy‐dispersive X‐ray spectroscopy (EDS) measurement was conducted (Figure S5, Supporting Information). The EDS characterization reveals that no In^3+^ ions is detected, and the atomic percentages of Cs, Cu, and I atoms are 29.34%, 20.3%, and 50.36%, respectively, which is in good agreement with the stoichiometry. The absence of In^3+^ is consistent with previous theoretical and experimental study, which reveals that the Cs_2_CuIn*X*_6_ compound is energetically unfavorable.^[^ ^30^ ^]^

### 2.2 Extending the Synthesis to Other Cs_3_Cu_2_ *X*_5_ NCs

It should be noted that, Cs_3_Cu_2_Cl_5_ and Cs_3_Cu_2_Br_5_ NCs also can be synthesized using the same strategy by substitution of the metal iodides with metal chlorides and bromides. The TEM and HRTEM images of Cs_3_Cu_2_Cl_5_ (Figure S6a,b, Supporting Information) and Cs_3_Cu_2_Br_5_ (Figure S7a,b, Supporting Information) NCs show quasi‐spherical and well‐defined hexagonal shapes, respectively. The average diameters are in the range of 21–28 nm for Cs_3_Cu_2_Cl_5_ and 12–15 nm for Cs_3_Cu_2_Br_5_ NCs. The XRD patterns of Cs_3_Cu_2_Cl_5_ (Figure S6c, Supporting Information) and Cs_3_Cu_2_Br_5_ NCs (Figure S7c, Supporting Information) are in perfect agreement with the standard orthorhombic structure of their bulk counterparts, indicating that the resulting NCs have a high phase purity. Furthermore, elemental analyses via EDS confirm the 3:2:5 atomic ratio for Cs_3_Cu_2_Cl_5_ (Figure S6d, Supporting Information) and Cs_3_Cu_2_Br_5_ (Figure S7d, Supporting Information) NCs, and no In^3+^ ion is present.

### 2.3 Optical Properties of Cs_3_Cu_2_ *X*_5_ NCs

**Figure 2:**
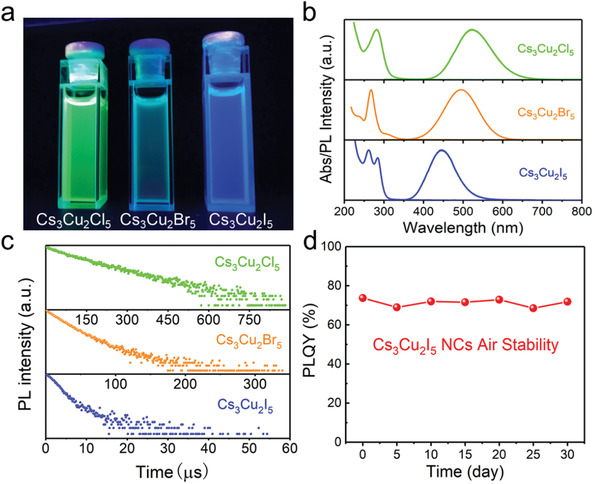
a) Photograph of colloidal solution of Cs_3_Cu_2_ *X*_5_ (*X* = Cl, Br, I) NCs in hexane under UV light (λ = 254 nm). b) Normalized absorption and PL spectra, and c) TRPL decay dynamics of the Cs_3_Cu_2_ *X*_5_ NCs. d) PLQY stability of Cs_3_Cu_2_I_5_ NCs in ambient atmosphere.

We further investigated the photophysical properties of Cs_3_Cu_2_ *X*_5_ (*X* = Cl, Br, I) NCs using steady‐state UV–vis absorption and emission spectroscopies, as well as time‐resolved PL (TRPL) decay dynamics. **Figure** **2**a shows the photograph of purified colloidal dispersions of Cs_3_Cu_2_ *X*_5_ NCs in hexane under UV lamp irradiation (254 nm). Highly luminescent green, sky blue, and deep blue emissions under UV light were observed for Cs_3_Cu_2_Cl_5_, Cs_3_Cu_2_Br_5_, and Cs_3_Cu_2_I_5_ NCs, respectively. The evolution of the PL peak with the change of the halide is contrary to that of the traditional lead halide perovskite NCs whose PL redshift as the halide composition changes from chloride to bromide and iodide.^[^ ^31^ ^]^ This interesting trend of the PL peak in Cs_3_Cu_2_ *X*_5_ NCs is consistent with the previous observations,^[^ ^22^, ^23^, ^27^ ^]^ which is related to the emission mechanism as discussed in the following sections. Figure 2b displays the absorption and emission spectra of the Cs_3_Cu_2_ *X*_5_ NCs. The emission spectrum of Cs_3_Cu_2_I_5_ NCs has a peak at 445 nm with a large Stokes shift of 161 nm and a full‐width at half‐maximum (FWHM) of 80 nm. It is worth noting that the PLQY of Cs_3_Cu_2_I_5_ NCs is 73.7%, which is, to the best of our knowledge, the highest PLQY among blue emission lead‐free perovskite or perovskite‐like NCs.^[^ ^24^, ^26^, ^32^, ^33^, ^34^, ^35^, ^36^, ^37^ ^]^ And comparison of optical properties of different lead‐free perovskite or perovskite‐like NCs with blue emission are summarized in Table S1, Supporting Information. The PL spectra of these Cs_3_Cu_2_ *X*_5_ NCs can be turned from 445 to 520 nm by adjusting their composition (Figure 2b) and have broad widths of 80–106 nm. These NCs exhibit remarkably large Stokes shifts of 161–238 nm, indicating no overlap between absorption and emission spectra. Such zero self‐absorption NCs are of great interest for scintillators and luminescent solar concentrators.^[^ ^38^, ^39^ ^]^ The TRPL measurements reveal bi‐exponential PL decay kinetics of the Cs_3_Cu_2_ *X*_5_ NCs with average lifetimes in the range of 1.92–95.79 µs, as shown in Figure 2c. The PLQYs of Cs_3_Cu_2_Cl_5_ (46.2%) and Cs_3_Cu_2_Br_5_ (3.9%) NCs are much lower than that of Cs_3_Cu_2_I_5_ NCs, indicating that the present general synthesis produces more defects in the chloride and bromide products. The detailed optical properties of Cs_3_Cu_2_ *X*_5_ NCs are summarized in **Table** **1**. It is worth noting that, in addition to a high PLQY, the Cs_3_Cu_2_I_5_ NCs also have excellent stability in air, as shown in Figure 2d, no noticeable degradation of PLQY over one month under ambient atmosphere. Such broadband, large Stokes shift combined with long lifetimes suggest that the PL emission of Cs_3_Cu_2_I_5_ NCs is likely originated from the STEs as those of low dimensional metal halides.^[^ ^28^, ^40^, ^41^, ^42^ ^]^

**Table 1:** Optical properties of Cs_3_Cu_2_ *X*_5_ (*X* = Cl, Br, I) NCs at room temperature

Nanocrystals	Abs. [nm]	PL peak [nm]	FWHM [nm]	Stokes shift [nm]	τ_ave._ [µs]	PLQY
Cs_3_Cu_2_Cl_5_	282	520	106	238	95.79	46.2%
Cs_3_Cu_2_Br_5_	267	495	101	228	16.36	3.9%
Cs_3_Cu_2_I_5_	284	445	80	161	1.92	73.7%

### 2.4 Photophysics of Cs_3_Cu_2_I_5_ NCs

**Figure 3:**
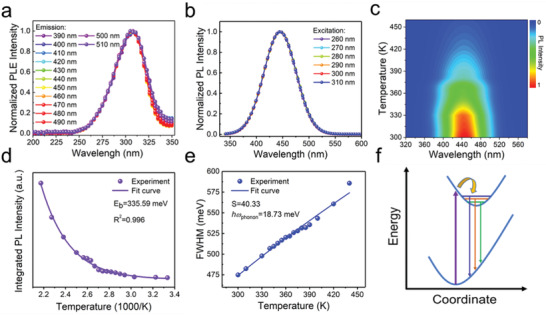
a) PLE spectra and b) PL spectra of Cs_3_Cu_2_I_5_ NCs measured at different emission and excitation wavelengths, respectively. c) Pseudocolor map of temperature‐dependent PL spectra of the Cs_3_Cu_2_I_5_ NCs. d) The correlation between integrated PL intensity and temperature derived from (c). By fitting the curve, the exciton binding energy was extracted. e) The fitting results of the FWHM as a function of temperature derived from (c). f) The coordinate diagram demonstrating the photophysical process in Cs_3_Cu_2_I_5_ NCs.

To further investigate the intrinsic emission mechanism, emission wavelength‐dependent PLE spectra and excitation wavelength‐dependent and temperature‐dependent PL spectra were carried out (**Figure** **3**a–c). Because of the similar properties of Cs_3_Cu_2_ *X*_5_ NCs, we take Cs_3_Cu_2_I_5_ NCs as the example in the following context. As shown in Figure 3a, when the monitoring emission was changed from 390 to 510 nm, the normalized PLE spectra showed identical shapes. Similarly, when the excitation was varied from 260 to 310 nm, the normalized PL spectra exhibited the same features as well (Figure 3b). Such identical features of PLE and PL spectra at different wavelengths indicate that the blue emission originates from the recombination of the same excited state instead of ion luminescence.^[^ ^43^, ^44^, ^45^ ^]^ This supports that STE is the emission mechanism of Cs_3_Cu_2_I_5_ NCs. Figure 3c shows the temperature‐dependent PL pseudocolor map of Cs_3_Cu_2_I_5_ NCs. The PL intensity decreases monotonously, and simultaneously, the FWHM broadens significantly with increasing temperature, both of which indicate that more phonons coupling with excitons occur at higher temperatures that activate nonradiative recombination process.^[^ ^37^, ^43^, ^46^ ^]^ Figure 3d,e shows plots of temperature‐dependent integrated PL intensity and FWHM of Cs_3_Cu_2_I_5_ NCs, respectively. The exciton binding energy (*E*_b_) of Cs_3_Cu_2_I_5_ NCs can be derived from the Equation (1):

(1) $$I\left(T\right)\;=\frac{I_{0}}{1+A\text{exp}\left(-\frac{E_{\text{b}}}{k_{\text{B}}T}\right)}$$

where *I*_0_ is the integrated PL intensities at 0 K, *A* is a proportional coefficient, and *k*_B_ is the Boltzmann constant. By fitting the curve in Figure 3d, the *E*_b_ of Cs_3_Cu_2_I_5_ NCs is calculated to be 335.59 meV, which is much larger than that of conventional 3D perovskite NCs (≈18 meV).^[^ ^47^ ^]^ Such a high *E*_b_ is attributed to the 0D electronic structure of Cs_3_Cu_2_I_5_ NCs. In this 0D structure, photogenerated excitons are strongly confined in individual [Cu_2_I_5_]^3−^ clusters, resulting in a high *E*_b_.^[^ ^48^ ^]^ Moreover, the exciton–phonon coupling in Cs_3_Cu_2_I_5_ NCs is reflected by the Huang–Rhys factor (*S*) and the phonon frequency (*ℏω*_phonon_), which can be extracted by fitting the temperature‐dependent FWHM curve using the following Equation (2):

(2) $$\text{FWHM}\;=\;2.36\sqrt{S}\hbar \omega_{\text{phonon}}\sqrt{\coth\frac{\hbar \omega_{\text{phonon}}}{2k_{\text{B}}T}}$$

The *S* factor and *ℏω*_phonon_ are calculated to be 40.33 and 18.73 meV, respectively. It is worth noting that the value of *ℏω*_phonon_ agrees well with that of fully‐inorganic perovskite NCs (≈16–40 meV).^[^ ^37^, ^49^, ^50^, ^51^ ^]^ More importantly, the Huang–Rhys factor *S* of Cs_3_Cu_2_I_5_ NCs is relatively larger than that of most reported common emissive NCs, like CdSe (1),^[^ ^52^ ^]^ ZnSe (0.31),^[^ ^53^ ^]^ and CsPbBr_3_ (3.22).^[^ ^54^ ^]^ This strong exciton–phonon coupling indicates that the Cs_3_Cu_2_I_5_ NCs have a soft crystal lattice and it is easy to induce the formation of self‐trapped excited states.^[^ ^44^, ^55^ ^]^

Based on the above discussions, it is obvious that the STEs dominate the emission mechanism of Cs_3_Cu_2_I_5_ NCs. The exciton self‐trapping processes for Cs_3_Cu_2_I_5_ NCs can be depicted in the coordinate diagram, as shown in Figure 3f. Upon photoexcitation, electrons are excited from ground state to the high energy free‐exciton excited state, which can induce the formation of self‐trapped excited state due to the lattice deformation driven by strong electron–phonon coupling, and immediately, the excited electrons undergo ultrafast relaxation and intersystem crossing process from free‐exciton excited state to self‐trapped excited state, finally generating a bright and broadband blue emission with a large Stokes shift and a long lifetime (1.92 µs).^[^ ^28^, ^40^, ^56^, ^57^ ^]^

Considering the similarity of Cs_3_Cu_2_ *X*_5_, the Cs_3_Cu_2_Cl_5_, and Cs_3_Cu_2_Br_5_ NCs should hold the same emission mechanism with the Cs_3_Cu_2_I_5_ NCs. The unconventional evolution of PL peak with the change of the halide (Figure 2b) can be attributed to the STE‐based emission in Cs_3_Cu_2_ *X*_5_ NCs. Since the lattice of Cs_3_Cu_2_ *X*_5_ deforms upon the photoexcitation, the PL peak is determined by the distorted structure. The PL peak energy is given by *E*_g_–*E*_b_, where *E*_g_ is the bandgap. A theoretical calculation shows that the ground state bandgaps of Cs_3_Cu_2_ *X*_5_ follows the same trend with that of lead halide perovskites,^[^ ^27^ ^]^ that is, the ground state bandgaps of Cs_3_Cu_2_ *X*_5_ decrease when the halide changes from chloride to bromide and iodide. However, the lattice deformation might induce significant changes of *E*_g_ and *E*_b_ and the amount of change depends on the extent of deformation. Recently, Gautier et al. showed that halogen plays an important role on the exciton self‐trapping and they found the self‐trapping depth follows the trend Cl > Br > I, which is attributed to the different deformation potentials of the lattice induced by different M–X bonding.^[^ ^58^ ^]^ Therefore, it is possible that the smaller halide ion induces larger lattice distortion and thus larger changes of *E*_g_ and *E*_b_, resulting in the unconventional evolution of PL peak in Cs_3_Cu_2_ *X*_5_ NCs.

### 2.5 Radioluminescence in Cs_3_Cu_2_I_5_ NCs

**Figure 4:**
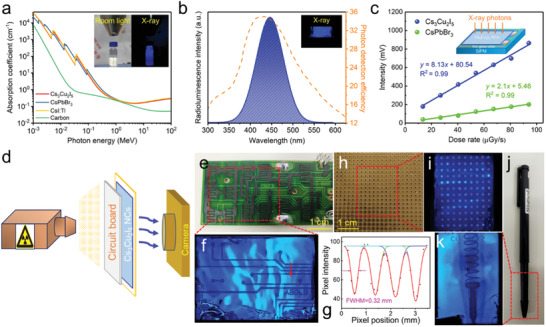
a) The X‐ray absorption coefficients of Cs_3_Cu_2_I_5_, CsPbBr_3_, CsI:Tl and carbon dots as a function of photon energy. The inset shows the photographs of colloidal solution of Cs_3_Cu_2_I_5_ NCs under room light and X‐ray irradiation with the energy of 50 keV. b) The RL spectrum of NCs film under 50 keV X‐ray excitation and the curve of photon detection efficiency of SiPM. The inset shows the photograph of the Cs_3_Cu_2_I_5_ film under X‐ray. c) The response intensity of Cs_3_Cu_2_I_5_ NCs and CsPbBr_3_ NCs as a function of X‐ray dose rate. The inset schematically shows the measurement, in which the NC films on glass are coupled with SiPM. d) Schematic of the prototype projection system for X‐ray imaging, and the sequence is an X‐ray source, a circuit board, Cs_3_Cu_2_I_5_ NC scintillators and a smartphone camera. e) The photograph and f) the corresponding X‐ray image of a circuit board. g) Point spread function (red arrow in (f)) of the intensity profile is fitted with Gaussian function, and the FWHM is obtained as the spatial resolution. h) The photograph and i) X‐ray image of a universal board. j) The photograph and k) X‐ray image of a ball‐point pen with an encapsulated metallic spring.

The high PLQY, broadband blue emission, and zero self‐absorption make the Cs_3_Cu_2_I_5_ NCs promising candidates for scintillators. To testify their scintillation performance, we first compare the absorption coefficient (α) of the Cs_3_Cu_2_I_5_ NCs as a function of photon energies with three conventional scintillators, such as CsPbBr_3_ NCs, CsI:Tl crystal, and carbon dots, as shown in **Figure** **4**a. The absorption coefficient (or the X‐ray stopping power), α, of a material is closely related to the effective atomic number *Z*_eff_, with the relation *α* ∝ *ρZ*_eff_^4^/*E* ^3^, where ρ is mass density and *E* is the X‐ray photon energy.^[^ ^9^, ^15^, ^19^ ^]^ The *Z*_eff_ and ρ of Cs_3_Cu_2_I_5_ are 52.4 and 4.28 g cm^−3^, respectively. The absorption coefficient of the Cs_3_Cu_2_I_5_ NCs across the medical radiography window (18–30 keV) is much higher than that of carbon dots, comparable to that of CsI:Tl crystal and slightly lower than that of CsPbBr_3_ NCs, which is consistent with the sequence of their *Z*_eff_.^[^ ^19^ ^]^

Both the colloidal Cs_3_Cu_2_I_5_ NC solution and film show bright blue RL under X‐ray excitation (insets in Figure 4a,b), suggesting promising RL performance of the Cs_3_Cu_2_I_5_ NCs. The mechanism of RL in Cs_3_Cu_2_I_5_ NCs can be explained by photoelectric ionization effect, as previously reported in CsPb*X*_3_ NCs scintillators.^[^ ^15^ ^]^ Upon X‐ray excitation, a large number of electrons are ejected from lattice atoms of Cs_3_Cu_2_I_5_ NCs via photoelectric ionization, which can generate abundant energetic electrons and holes. Subsequently, the energetic electrons induce the generation of high‐energy secondary electrons. Those generated hot carriers then undergo thermal relaxation process and produce STEs. Finally, radiative recombination of STEs produce RL.^[^ ^15^ ^]^

For sensitive X‐ray detection, the RL is required to match the response peaks of photodetectors.^[^ ^2^ ^]^ The RL of our Cs_3_Cu_2_I_5_ NCs is shown in Figure 4b, which was measured under X‐ray irradiation with an energy of 50 keV. Obviously, the RL spectrum matches the photon detection efficiency curve of a commercial SiPM (dotted line in Figure 4b) quite well, which is an important advantage of Cs_3_Cu_2_I_5_ NCs as scintillators. It should be noted that the RL spectrum is consistent with the PL spectrum of Cs_3_Cu_2_I_5_ NCs, indicating both of the luminescence originate from the identical radiative recombination path.

We used a configuration shown in the inset of Figure 4c, in which the NC scintillators were coupled with a commercial SiPM, to measure the response of Cs_3_Cu_2_I_5_ NC scintillators to different X‐ray dose rate. In the measured range of dose rate (13.4–94.1 µGy s^−1^), the Cs_3_Cu_2_I_5_ NC scintillators show linear response to X‐ray dose rate, which is similar to that of CsPbBr_3_ NC scintillators (Figure 4c). However, the response of Cs_3_Cu_2_I_5_ NCs is higher than that of CsPbBr_3_ NCs under different X‐ray dose rate, implying a higher light yield for Cs_3_Cu_2_I_5_ NCs. To obtain the light yield of Cs_3_Cu_2_I_5_ NC scintillators, we use CsPbBr_3_ NCs as the reference and apply the following Equation (3):^[^ ^19^ ^]^

(3) $$\frac{\text{RL}\;{\text{LY}}_{{\text{Cs}}_{\text{3}}{\text{Cu}}_{\text{2}}{\text{I}}_{\text{5}}}}{\text{RL}\;{\text{LY}}_{{\text{CsPbBr}}_{\text{3}}}}\;=\;\frac{A_{{\text{Cs}}_{\text{3}}{\text{Cu}}_{\text{2}}{\text{I}}_{\text{5}}}}{A_{{\text{CsPbBr}}_{\text{3}}}}\;\times \;\frac{\int I_{{\text{CsPbBr}}_{\text{3}}}\left(\lambda \right)S\left(\lambda \right)\text{d}\lambda /\int I_{{\text{CsPbBr}}_{\text{3}}}\left(\lambda \right)\text{d}\lambda }{\int I_{{\text{Cs}}_{3}{\text{Cu}}_{2}{\text{I}}_{\text{5}}}\left(\lambda \right)S\left(\lambda \right)\text{d}\lambda /\int I_{{\text{Cs}}_{3}{\text{Cu}}_{2}{\text{I}}_{\text{5}}}\left(\lambda \right)\text{d}\lambda }$$

where *A* is the corrected response amplitude of scintillator, *I*(λ) and *S*(λ) are the corrected wavelength dependent RL spectra and detection efficiency of SiPM, respectively. The light yield of CsPbBr_3_ NC scintillators was measured to be 21 000 photons per MeV by Zhang et al.^[^ ^16^ ^]^ The response intensity of Cs_3_Cu_2_I_5_ NCs is close to four times higher than that of CsPbBr_3_ NCs. By correcting the RL spectra of the two NCs based the detection efficiency of the SiPM, we could calculate the light yield of Cs_3_Cu_2_I_5_ NCs to be ≈79 279 photons per MeV. Such high light yield is superior to some conventional efficient scintillators, such as CsI:Tl (54 000 photons per MeV) and LYSO:Ce (33 200 photons per MeV), as shown in Figure S8, Supporting Information.

The high light yield of Cs_3_Cu_2_I_5_ NCs might originate from their emission mechanism, that is, STE‐based emission, in addition to the high PLQY. In a scintillator, the absorption of a high energy X‐ray photon induces a large number of excitons.^[^ ^59^ ^]^ For the lead halide NCs, the emission originates from band‐to‐band recombination, thus an individual nanoparticle is a single emission center. The multiple excitons generated by a X‐ray photon in a lead halide perovskite NC lead to a high probability of Auger recombination, resulting in radioluminescence with a decreased intensity. For Cs_3_Cu_2_I_5_ NCs, an individual nanoparticle can have many emission centers, because multiple local lattice distortions can occur, leading to multiple trapped excitons. Furthermore, the multiple self‐trapped excitons are localized and separated by the 0D structure of Cs_3_Cu_2_I_5_, avoiding the Auger recombination. Therefore, the Cs_3_Cu_2_I_5_ NCs have a much higher light yield than the lead halide perovskite NCs.

The Cs_3_Cu_2_I_5_ NCs combine the advantages of large X‐ray absorption coefficient, high RL light yield, zero self‐absorption, perfect match with the response peak of SiPM, and solution‐processability, rendering Cs_3_Cu_2_I_5_ NCs as highly sensitive scintillators for X‐ray detection and imaging. To demonstrate the potential of Cs_3_Cu_2_I_5_ NC scintillators for X‐ray imaging, we built a prototype projection system, in which an X‐ray source, the imaging object, Cs_3_Cu_2_I_5_ NC film on slide glass and a smartphone camera were placed in sequence, as shown in Figure 4d and Figure S9, Supporting Information. As shown in Figure 4f, the conductive tracks in a circuit board can be clearly identified in the image collected by our prototype system. Additionally, the spatial resolution was obtained by fitting the point spread function of the intensity profile, which is 0.32 mm (Figure 4e–g). The photograph and X‐ray image of a universal board are shown in Figure 4h,i. Holes in the universal board are clearly visible. The projection system also clearly reveals the spring inside a ball‐point pen as shown in Figure 4j,k.

## 3 Conclusion

In summary, highly luminescent, reabsorption‐free are demonstrated to be efficient X‐ray scintillators. The Cs_3_Cu_2_I_5_ NCs exhibit a PLQY of 73.7% which is a new record for blue emission lead‐free perovskite or perovskite‐like NCs. The sensitive Cs_3_Cu_2_I_5_ NC scintillators originate from the high PLQY, zero self‐absorption, and perfect match between PL peak and the response peak of SiPM. These NC scintillators have similar X‐ray stopping power with CsI:Tl, and exhibit a high light yield of ≈79 279 photons per MeV, which is higher than CsPbBr_3_ NCs and most of conventional scintillators, such as CsI:Tl and LYSO:Ce. The capability of X‐ray imaging was evidenced by prototype experiments adopting solution‐processed Cs_3_Cu_2_I_5_ NC films. The present work demonstrates that NCs with STE emission might be promising scintillator for low cost, large area, and flexible X‐ray imaging.

## 4 Experimental Section

##### Chemicals

Cuprous chloride (CuCl, ≥99.95%), cuprous bromide (CuBr, 99.99%), cuprous iodide (CuI, 99.95%), cesium carbonate (Cs_2_CO_3_, 99.9%), and OLA (tech. grade, 70%) were purchased from Aladdin. Indium (III) chloride (InCl_3_ anhydrous, 99.99%), indium (III) bromide, (InBr_3_ anhydrous, 99.99%), indium (III) iodide, (InI_3_ anhydrous, 99.999%), OA (tech. grade, 90%), and 1‐octadecene (ODE, tech. grade, 90%) were purchased from Alfa Aesar. Ethyl acetate was purchased from Greagent. Hexane (≥97%) was purchased from Sinopharm Chemical Reagent. All the chemicals were used as received without further purification.

##### Preparation of Cs‐Oleate

Cs_2_CO_3_ (5 mmol), OA (5 mL), and ODE (20 mL) were loaded into a 100 mL three‐neck flask and dried for 1 h at 120 °C, and then heated to 150 °C under N_2_ atmosphere until all Cs_2_CO_3_ reacted with OA and ODE. Since Cs‐oleate precipitated out of ODE at room‐temperature, it had to be preheated to 120 °C before injection.

##### Synthesis of Cs_3_Cu_2_ *X*_5_ (*X* = Cl, Br, I) NCs

In a typical synthesis of Cs_3_Cu_2_I_5_ NCs, CuI (0.8 mmol), InI_3_ (0.4 mmol), ODE (10 mL), OA (0.8 mL), and OLA (0.8 mL) were mixed in a three‐neck flask and degassed for 1 h at 120 °C under vacuum and heated to 180 °C under N_2_ and Cs‐oleate solution (2 mL, 0.4 m) was quickly injected; 20 s later, the reaction mixture was cooled by the ice‐water bath. In a typical synthesis of Cs_3_Cu_2_Cl_5_ NCs, CuCl (0.2 mmol), InCl_3_ (0.4 mmol), ODE (5 mL), OA (1 mL), and OLA (1 mL) were mixed in a three‐neck flask and degassed for 1 h at 120 °C under vacuum and heated to 180 °C under N_2_ and Cs‐oleate solution (0.75 mL, 0.4 m) was swiftly injected; 20 s later, the reaction mixture was cooled by the ice‐water bath. In a typical synthesis of Cs_3_Cu_2_Br_5_ NCs, CuBr (0.2 mmol), InBr_3_ (0.3 mmol), ODE (6 mL), OA (1 mL), and OLA (1 mL) were mixed in a small glass bottle, then heated to 200 °C under stirring and maintained for 30 min; next, Cs‐oleate solution (0.75 mL, 0.4 m) was quickly injected; 20 s later, the reaction mixture was cooled by the ice‐water bath. Addition of ethyl acetate to the crude solution (2:1 by volume) was followed by centrifugation at 8000 rpm for 3 min and the supernatant was discarded. The precipitate was dispersed in 5 mL of hexane. After the second centrifugation at 8000 rpm for 3 min, the supernatant was collected for characterization.

##### Preparation of Cs_3_Cu_2_I_5_ NC Films

The NC films were deposited on slide glass substrates. The slide glasses were cleaned in a diluted detergent solution for 15 min, followed by using subsequent sonication in deionized water, acetone, ethanol, and isopropanol for 15 min, respectively. After drying with a N_2_ flow, the substrates were transferred in an oven and baked at 60 °C for 30 min. The Cs_3_Cu_2_I_5_ NC film was fabricated by drop casting Cs_3_Cu_2_I_5_ NCs supernatant (≈50 mg mL^−1^) onto a slide glass substrate. The film was formed under ambient condition by slow evaporation of hexane in a fume hood. In order to obtain thick film, the above depositions needed to be repeated for 10 times.

##### Characterization

TEM images were obtained using a FEI Tecnai G2 F30 transmission electron microscope in EFTEM mode operated at 300 kV equipped with Scanning Transmission Electron Microscopy Energy Dispersion Spectrum (STEM‐EDS) and EDS mapping. XRD patterns were recorded on X'Pert PROX‐ray diffractometer from PANalytical B.V. equipped with Cu Kα radiation. Optical absorption spectra were collected using a Shimadzu UV‐3600 plus spectrophotometer. Excitation and emission spectra were collected using a Zolix OmniFluo spectrofluorometer. The absolute PLQYs of the Cs_3_Cu_2_ *X*_5_ (*X* = Cl, Br, I) NCs were determined using an Edinburgh FLS980 spectrofluorometer with a calibrated integrating sphere. Samples were excited at a wavelength of 280 nm using a xenon lamp source. TRPL decay of Cs_3_Cu_2_ *X*_5_ (*X* = Cl, Br, I) NCs were recorded with time‐correlated single‐photon counting technique on an Edinburgh FLS980 phosphorescence lifetime system and excited at a wavelength of 280 nm. The temperature‐dependent PL spectrum measurements were recorded using a Zolix OmniFluo spectrofluorometer and excited at a wavelength of 280 nm using a xenon lamp source with temperature ranging from 300 to 460 K using a liquid helium cooler. The RL of Cs_3_Cu_2_I_5_ NCs was characterized using an X‐ray tube (M237, Newton Scientific) at 50 kV and a fiber optic spectrometer (Ocean Optics USB 2000+). The X‐ray response intensity was tested by a silicon photomultiplier (SiPM) (JSP‐TN3050‐SMT) and the data was collected by an oscilloscope (Keysight).

## Conflict of Interest

The authors declare no conflict of interest.

## Supporting information

Supporting Information
