# Supplementary material for: Efficient and Reabsorption‐Free Radioluminescence in Cs3Cu2I5 Nanocrystals with Self‐Trapped Excitons
Source: Adv Sci (Weinh). 2020 Apr 16;7(11):2000195. doi: 10.1002/advs.202000195 (PMC7284214; doi:10.1002/advs.202000195)
Supplement: Supplementary file 1 — Supporting Information [file ADVS-7-2000195-s001.pdf]

Copyright WILEY-VCH Verlag GmbH & Co. KGaA, 69469 Weinheim, Germany, 2020.

ADVANCED SCIENCE
Open Access

Supporting Information

for *Adv. Sci.,* DOI: 10.1002/advs.202000195

Efficient and Reabsorption-Free Radioluminescence in $Cs_3Cu_2I_5$ Nanocrystals with Self-Trapped Excitons

*Linyuan Lian, Moyan Zheng, Weizhuo Zhang, Lixiao Yin, Xinyuan Du, Peng Zhang, Xiuwen Zhang, Jianbo Gao, Daoli Zhang, Liang Gao, Guangda Niu, Haisheng Song, Rong Chen, Xinzheng Lan, Jiang Tang, and Jianbing Zhang**

Copyright WILEY-VCH Verlag GmbH & Co. KGaA, 69469 Weinheim, Germany, 2020.

Supporting Information

Efficient and Reabsorption-Free Radioluminescence in $Cs_3Cu_2I_5$ Nanocrystals with Self-Trapped Excitons

*Linyuan Lian, Moyan Zheng, Weizhuo Zhang, Lixiao Yin, Xinyuan Du, Peng Zhang, Xiuwen Zhang, Jianbo Gao, Daoli Zhang, Liang Gao, Guangda Niu, Haisheng Song, Rong Chen, Xinzheng Lan, Jiang Tang, Jianbing Zhang**

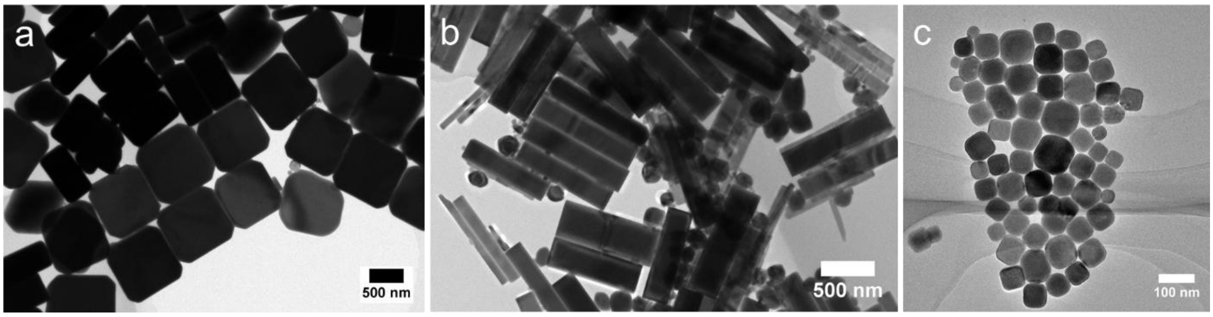

Figure S1. TEM images of (a) $Cs_3Cu_2Cl_5$, (b) $Cs_3Cu_2Br_5$, and (c) $Cs_3Cu_2I_5$, without the addition of InX_3, the $Cs_3Cu_2X_5$ NCs grew too large and loss colloidal stability in solvents.

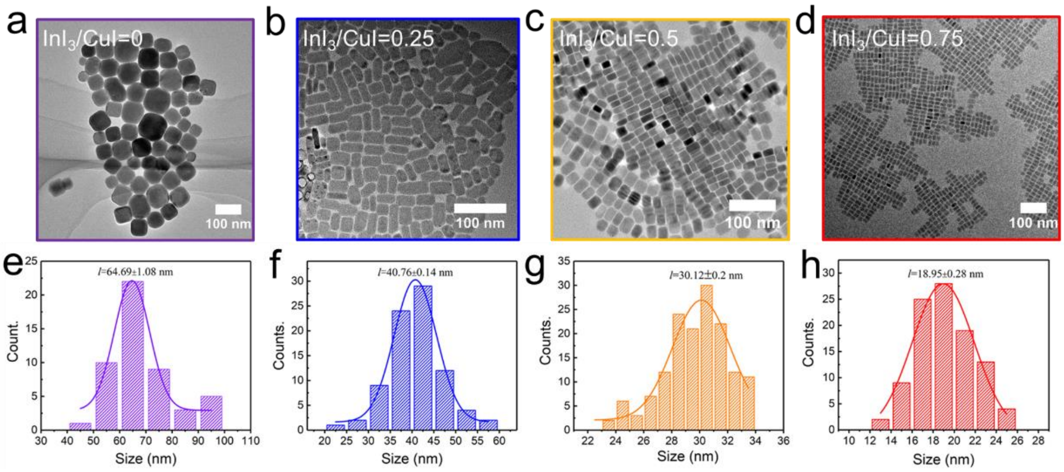

Figure S2. The obtained TEM images of $Cs_3Cu_2I_5$ NCs by changing the InI_3-to-CuI molar ratio in the reactant, InI_3/CuI= (a) 0, (b) 0.25, (c) 0.5, (d) 0.75. The corresponding size distribution of TEM images of (a-b) is shown in (e-h).

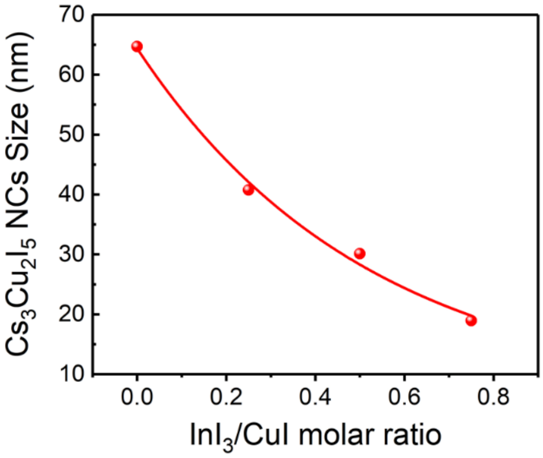

Figure S3. Dependence of the $Cs_3Cu_2I_5$ NCs size on the InI_3-to-CuI molar ratio in the reactant.

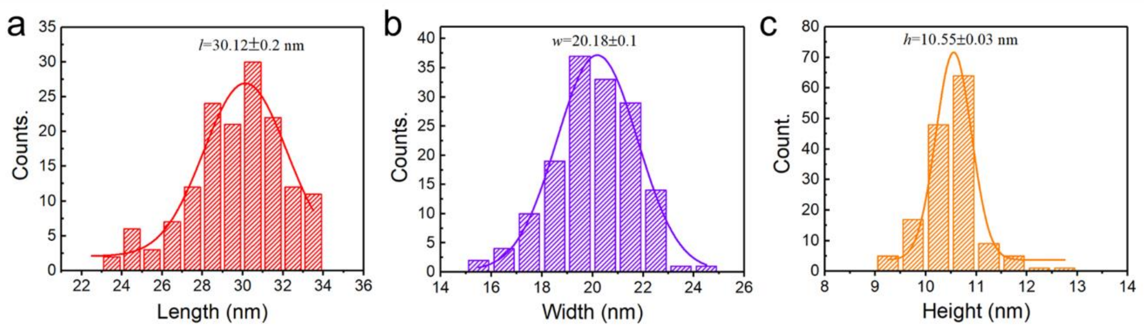

Figure S4. Distributions of three characteristic dimensions of $Cs_3Cu_2I_5$ NCs. (a) the side length, *l*=30.12±0.2 nm; (b) the side width, *w*=20.18±0.1 nm; (c) the height, *h*=10.55±0.03 nm.

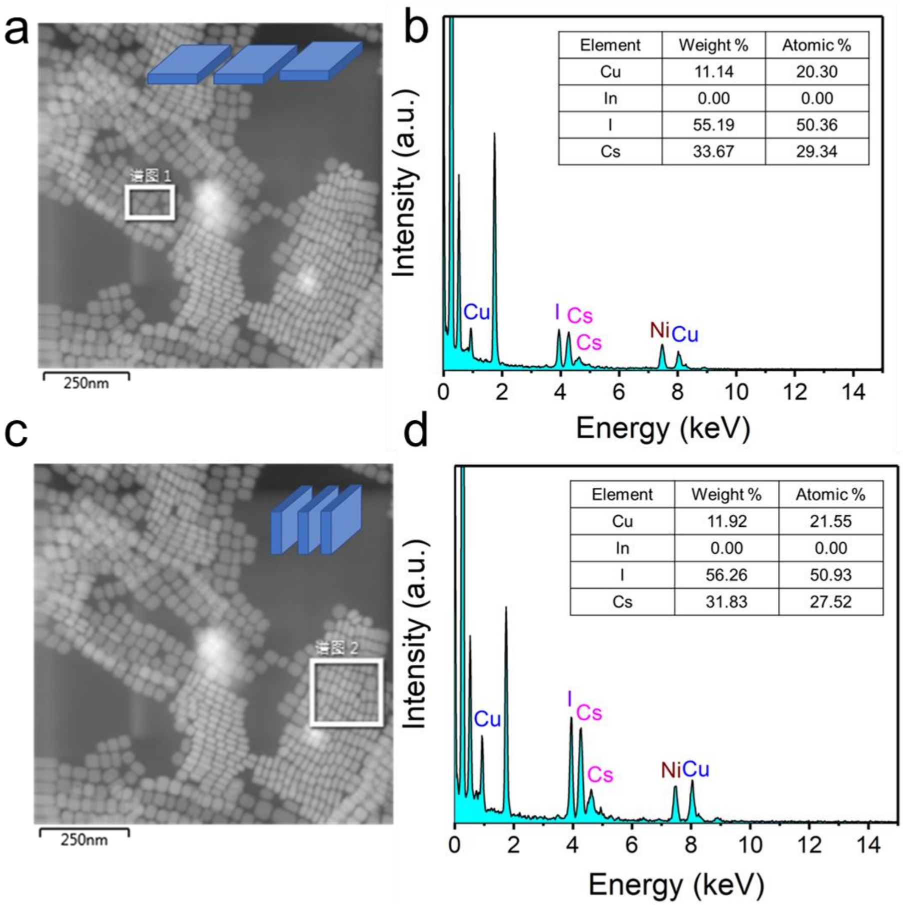

Element	Weight %	Atomic %
Cu	11.14	20.30
In	0.00	0.00
I	55.19	50.36
Cs	33.67	29.34

Element	Weight %	Atomic %
Cu	11.92	21.55
In	0.00	0.00
I	56.26	50.93
Cs	31.83	27.52

Figure S5. (a) HADDF-STEM image of $Cs_3Cu_2I_5$ NCs lying flat on grid and (b) the corresponding EDS spectrum and elemental analysis of $Cs_3Cu_2I_5$ NCs. (c) HADDF-STEM image of the cross-section of $Cs_3Cu_2I_5$ NCs by face-to-face manner on grid and (d) the corresponding EDS spectrum and elemental analysis of $Cs_3Cu_2I_5$ NCs.

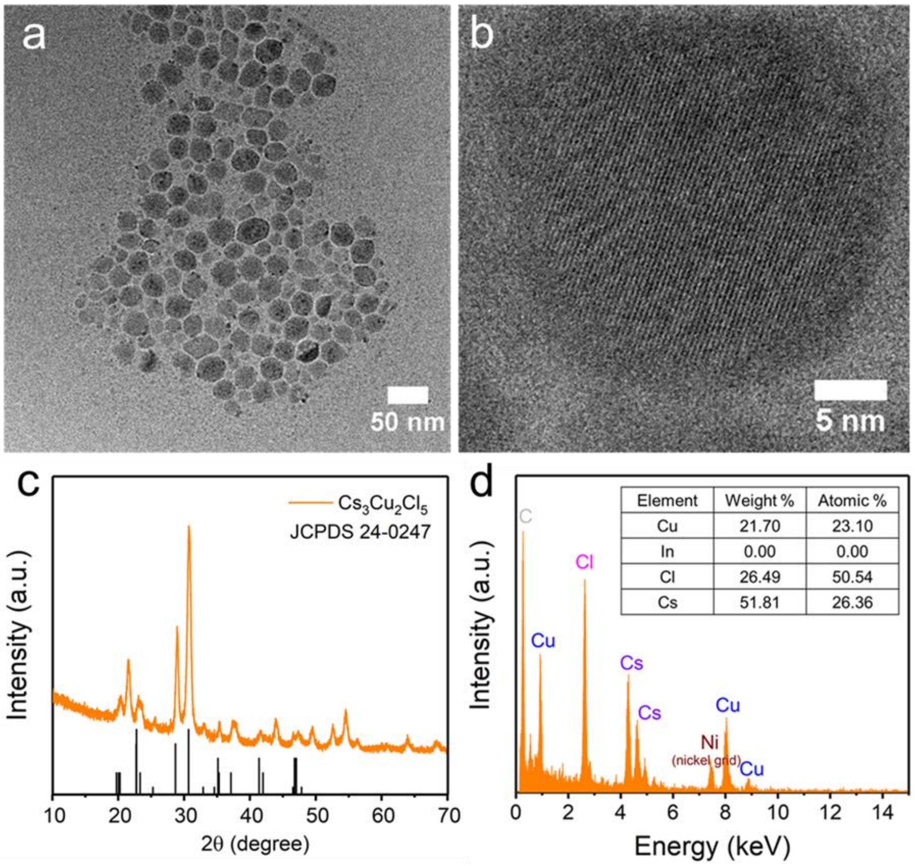

Figure S6. (a) TEM and (b) HRTEM images of $Cs_3Cu_2Cl_5$ NCs. (c) XRD pattern and (d) EDS spectrum and elemental analysis of $Cs_3Cu_2Cl_5$ NCs. We note that to avoid copper interference, we use nickel grid.

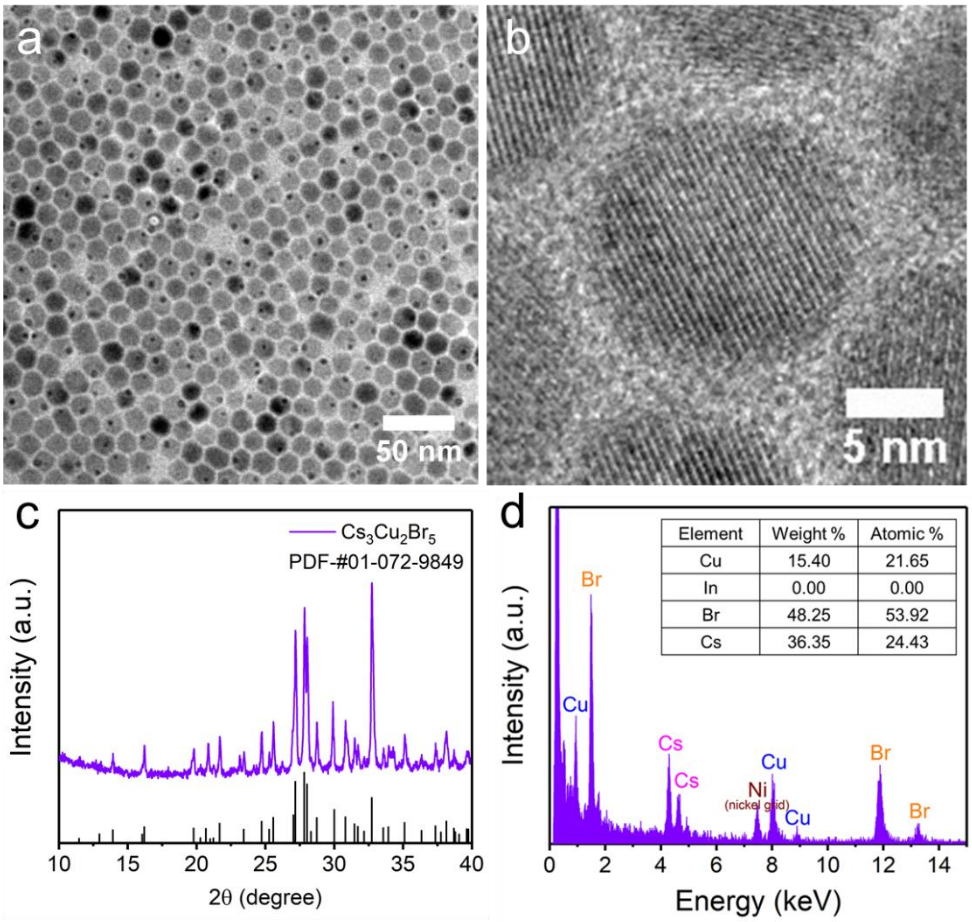

Figure S7. (a) TEM and (b) HRTEM images of $Cs_3Cu_2Br_5$ NCs. (c) XRD pattern and (d) EDS spectrum and elemental analysis of $Cs_3Cu_2Br_5$ NCs.

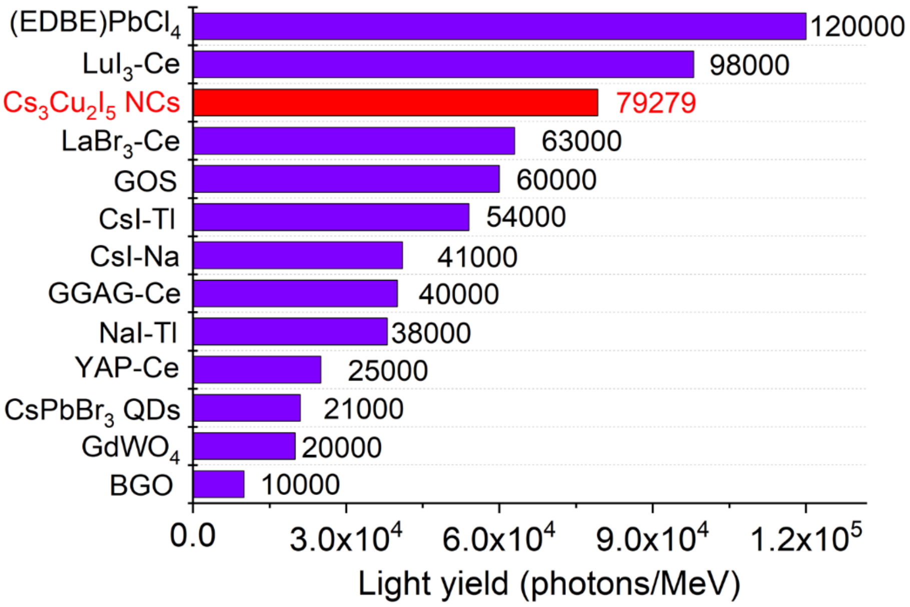

Figure S8. Light yield comparison of the $Cs_3Cu_2I_5$ NCs and some conventional scintillators.[1-3]

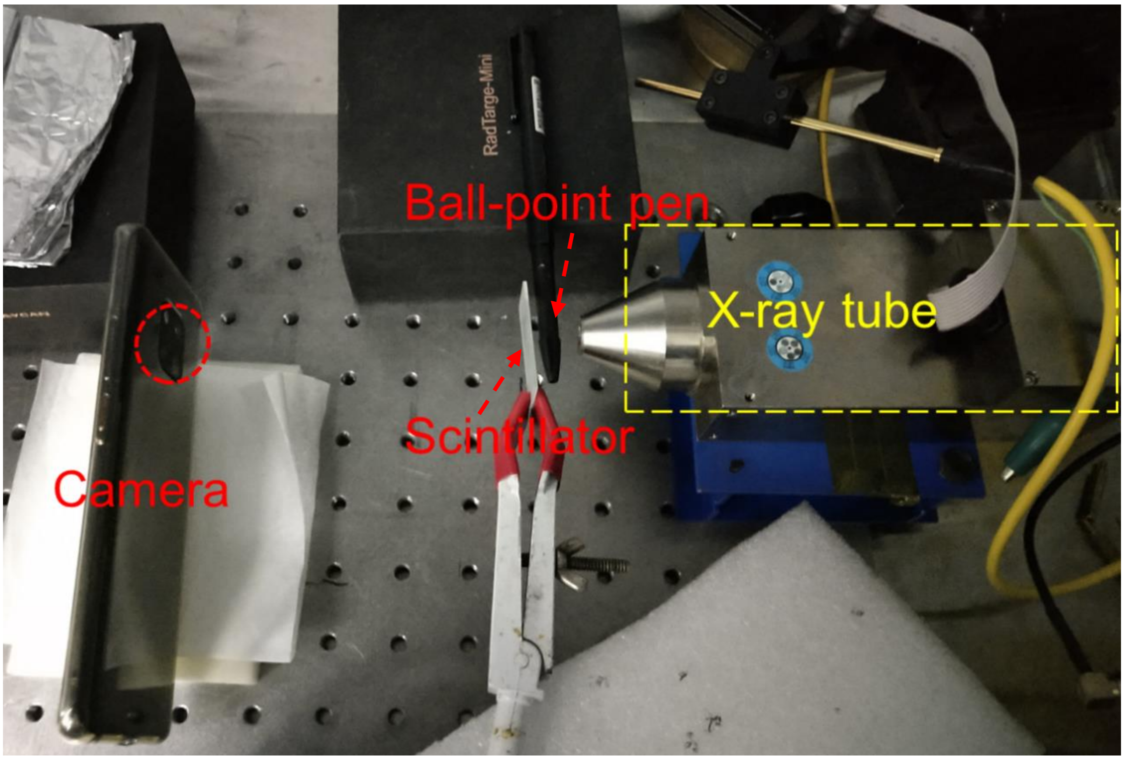

Figure S9. Prototype projection system for X-ray imaging.

Table S1. Optical Properties Comparison of Different Lead-Free Perovskite or Perovskite-Like NCs with Blue Emission.

Nanocrystals	PL peak (nm)	FWHM (nm)	Eb (meV)	PLQY (%)	Ref.
$MA_3Bi_2Br_9$	430	62		12	4
$Cs_3Bi_2Br_9$	460	45	67±5	4.5	5
$Cs_3Bi_2Br_9$	410	48	210.7	19.4	6
Cl-$MA_3Bi_2Br_9$	422	41	259.1	54.1	7
$Rb_7Bi_3Cl_{16}$	437	93		28.4	8
$Cs_3Sb_2Br_9$	410	41	548	46	9
$Cs_3Cu_2I_5$	441		371	67	10
$Cs_3Cu_2I_5$	444	79		35	11
$Cs_3Cu_2I_5$	445	80	335.59	73.7	This work

REFERENCES

[1] C. W. E. v. Eijk, *Phys. Med. Biol.* **2002**, 47, R85.

[2] Y. Zhang, R. Sun, X. Ou, K. Fu, Q. Chen, Y. Ding, L.-J. Xu, L. Liu, Y. Han, A. V. Malko, X. Liu, H. Yang, O. M. Bakr, H. Liu, O. F. Mohammed, *ACS Nano* **2019**, 13, 2520.

[3] M. D. Birowosuto, D. Cortecchia, W. Drozdowski, K. Brylew, W. Lachmanski, A. Bruno, C. Soci, *Sci Rep* **2016**, 6, 37254.

[4] M. Leng, Z. Chen, Y. Yang, Z. Li, K. Zeng, K. Li, G. Niu, Y. He, Q. Zhou, J. Tang, *Angew. Chem. Int. Ed.* **2016**, 55, 15012.

[5] B. Yang, J. Chen, F. Hong, X. Mao, K. Zheng, S. Yang, Y. Li, T. Pullerits, W. Deng, K. Han, *Angew Chem Int Ed Engl* **2017**, 56, 12471.

[6] M. Leng, Y. Yang, K. Zeng, Z. Chen, Z. Tan, S. Li, J. Li, B. Xu, D. Li, M. P. Hautzinger, Y. Fu, T. Zhai, L. Xu, G. Niu, S. Jin, J. Tang, *Adv. Funct. Mater.* **2018**, 28, 1704446.

[7] M. Leng, Y. Yang, Z. Chen, W. Gao, J. Zhang, G. Niu, D. Li, H. Song, J. Zhang, S. Jin, J. Tang, *Nano Lett.* **2018**, 18, 6076.

[8] J.-L. Xie, Z.-Q. Huang, B. Wang, W.-J. Chen, W.-X. Lu, X. Liu, J.-L. Song, *Nanoscale* **2019**, 11, 6719.

[9] J. Zhang, Y. Yang, H. Deng, U. Farooq, X. Yang, J. Khan, J. Tang, H. Song, *ACS Nano* **2017**, 11, 9294.

[10] P. Cheng, L. Sun, L. Feng, S. Yang, Y. Yang, D. Zheng, Y. Zhao, Y. Sang, R. Zhang, D. Wei, W. Deng, K. Han, *Angew Chem Int Ed Engl* **2019**, 58, 16087.

[11] P. Vashishtha, G. V. Nutan, B. E. Griffith, Y. Fang, D. Giovanni, M. Jagadeeswararao, T. C. Sum, N. Mathews, S. G. Mhaisalkar, J. V. Hanna, T. White, *Chem. Mater.* **2019**, 31, 9003.
